# Bayesian-Based Probabilistic Risk Assessment of Fipronil in Food: A Case Study in Taiwan

**DOI:** 10.3390/toxics11080677

**Published:** 2023-08-07

**Authors:** Yu-Syuan Luo

**Affiliations:** 1 Institute of Food Safety and Health, College of Public Health, National Taiwan University, Taipei 10617, Taiwan; ysluo@ntu.edu.tw; 2 Master of Public Health Program, National Taiwan University, Taipei 10617, Taiwan; 3 Population Health Research Center, College of Public Health, National Taiwan University, Taipei 10617, Taiwan

**Keywords:** fipronil, Markov Chain Monte Carlo simulation (MCMC), exposure assessment, Bayesian benchmark dose (BBMD), health risk assessment

## Abstract

Fipronil, a broad-spectrum insecticide, is widely used in agriculture and veterinary practices. Fipronil-induced neurotoxicity and potential adverse effects on humans and aquatic organisms have raised health concerns. Monitoring programs have been implemented globally to assess fipronil residues in food, including fruits, vegetables, and animal products. However, previous exposure assessments have often focused on specific food categories or subsets of items, resulting in limited insights into the overall health risks. Additionally, the large number of non-detect fipronil residues in food has introduced uncertainties in exposure assessment. To address these issues, a probabilistic exposure assessment and dose-response analysis were adopted in this study, considering the sample distribution below the detection limit to better characterize uncertainties and population variability in health risk assessments. The estimated fipronil exposure to the general public ranges from 6.38 × 10^−6^ ± 0.00017 mg/kg/day to 9.83 × 10^−6^ ± 0.00034 mg/kg/day. Only one out of 200,000 simulated individuals had a fipronil dose exceeding the probabilistic reference dose (0.048 mg/kg/day, pRfD), which aims to protect 99% of the population with effects less than 10% extra risk. By incorporating uncertainties in exposure and dose-response data, a more comprehensive understanding of the health risks associated with fipronil exposure in the Taiwanese population has been achieved.

## 1. Introduction

Fipronil, a broad-spectrum phenylpyrazole insecticide, has been widely used in crop protection, pest control, and veterinary practice. Fipronil shows potent neurotoxicity to insects and aquatic organisms such as *Daphnia magna* [1], shrimps [2], and crustaceans [3] via the inhibition of the gamma-aminobutyric acid (GABA)-gated chloride channel, resulting in convulsions, paralysis, and death [4]. Fipronil exposure can induce oxidative stress, neurotoxicity, hepatotoxicity, and nephrotoxicity in rodents and humans [5,6,7]. Additionally, studies showed that maternal exposure to fipronil could be placentally transferred to the fetus, leading to adverse health effects in newborns [8]. The widespread agricultural uses and the toxic potential of fipronil have raised increasing public concerns about fipronil residues in food.

National regulatory agencies have routinely surveyed fipronil residues in food to ensure the consumer’s safety. The fipronil monitoring data in food have been reported in China [9], Europe Union [10], India [11], Japan [12], and Taiwan [13]. Aside from fruits and vegetables, chicken eggs and animal products have been included in the surveillance programs to monitor the misuse of non-approved veterinary drugs, such as fipronil, in poultry farms against red mites [14,15]. Fipronil contamination in eggs has been reported in Europe, South Korea, Hong Kong, and Taiwan since 2017 [16,17]. Even though the fipronil residual data in fruits, vegetables, and eggs have been widely collected, most exposure and risk assessments target a specific food category [18,19,20,21] or a specific set of food items [9,22]. Additionally, the detection rate of fipronil in food is usually less than 10%. A large number of non-detects can introduce significant uncertainties in exposure assessment, as the “true” concentrations for non-detects may range from zero to the detection limit. A comprehensive aggregate exposure assessment considering the distribution below the detection limit would better characterize the health risks caused by environmental chemicals.

To calculate the health risk, the aggregate exposure estimate is compared with the health reference value (i.e., acceptable daily intake (ADI) or reference dose (RfD)). Traditionally, the ADI is derived by dividing the point of departure value (POD) from a chronic animal study (i.e., a no observed adverse effect level (NOAEL) or a benchmark dose lower confidence limit (BMDL)) by a generic safety factor of 100 or a chemical-specific adjustment factor (CASF) to account for the intra- and inter-species variability [23,24]. Currently, the deterministic ADI of fipronil was set at 0.0002 mg/kg/day according to the evaluation performed by the Joint Meeting on Pesticide Residues (JMPR), based on a NOAEL of 0.019 mg/kg/day for neurotoxicity in Sprague Dawley CD rats and a safety factor of 100. However, the default safety factors are assumed to be conservative toward protecting public health, where the degree of uncertainty in the ADI is not quantified or unknown [25]. The National Academy of Sciences (NAS) recommended the use of a probabilistic reference dose (pRfD), defined as a “risk-specific dose” [26]. Analytical tools, such as APROBA-plus [27] and Bayesian Benchmark dose (BBMD) [28], have been developed to support the probabilistic dose-response assessments, yet only a few studies incorporated the probabilistic concept in dose-response modeling and risk characterization [29].

In this study, we aimed to conduct a comprehensive investigation into the health risks associated with fipronil exposure in Taiwan, using a traditional deterministic approach or a probabilistic approach. By adopting the probabilistic approach and reconstructing the sample distribution that falls below the detection limit, we were able to provide a more accurate characterization of the uncertainty and population variability in the exposure and health risk assessment. Based on our findings, we strongly advocate adopting probabilistic risk assessment as it offers a flexible tool that can effectively address various regulatory requirements.

## 2. Materials and Methods

### 2.1. Aggregate Dietary Exposure Assessment of Fipronil

#### 2.1.1. Fipronil Residues in Food

The fipronil residual concentration in food was gathered from two data sources in Taiwan. The fipronil residues in fruits (*n* = 4011) and vegetables (*n =* 15,509) were retrieved from a publicly available database (2018–2023), “Dataset of Pesticide Residue Investigation in Commercially Available Food Products”, as released by the Taiwan Food and Drug Administration (Available online: https://data.gov.tw/dataset/8935 accessed on 25 April 2023). The fipronil residues in chicken eggs from 2014 to 2021 were acquired from the Department of Health, New Taipei City Government, detailed elsewhere [13]. The number of total samples, detects, average concentration, and standard deviation of fipronil in detected samples are summarized in Table 1.

#### 2.1.2. Reconstruction of Fipronil Distributions Using Bayesian—Based MCMC Approach

We reconstructed the fipronil concentrations in food based on a modified Markov Chain Monte Carlo (MCMC) method as described by Suzuki et al. [30], using the “rstan” package (version 2.21.2) in R software (version 4.0.4). The R code is detailed in Supplementary Materials File S1. Briefly, the Stan model has 5 components: “data”, “transformed data”, “parameters”, “model”, and “generated quantities”. The “data component” specifies the number of observed samples (N_obs), the number of censored samples (N_cen), and the value of observed data (Y_obs). The “transformed data” component computes the minimum and maximum values of Y_obs. The “parameters” component defines geometric mean (GM), geometric standard deviation (GSD), reporting limit (RL), and the estimates for non-detected values (Y_cen).

The “model” component specifies the prior distributions for the declared parameters, which plays an important role in MCMC modeling. Among the detectable samples (*n* = 120), fipronil residue in food follows the lognormal distribution as determined by the Kolmogorov—Smirnov test (α = 0.05). The geometric standard deviation of the detected fipronil concentrations was 4.16. Therefore, we applied the lognormal distribution with GM of 4 and GSD of 2 for the prior distribution of GSD. For the food category with only one detectable sample, we added an extra detectable value at the detection limit (0.002 ppm) to successfully run the MCMC modeling. The normal distributions were used as priors for GM~(Y_obs_max/2, Y_obs_max/4) and RL~(Y_obs_min, Y_obs_min/5).

The “generated quantities” component calculated the posterior distribution of log(arithmetic) density for observed and censored data. The cumulative distribution function (CDF) was used to estimate the likelihood of censored data. Lastly, the arithmetic mean and standard deviation were calculated from the GM and GSD.

For the MCMC runs, we calculated 4 Markov chains with 25,000~50,000 iterations per chain. The ratio of inter-chain variance to intra-chain variance ($\hat{R}$) less than 1.1 was used to determine the convergence of 4 MCMC chains. For the MCMC runs reaching convergence, the mean distribution parameters (mean_est, std_est) from the last 50% of iterations were used to define the posterior lognormal distributions of fipronil residues in food.

#### 2.1.3. Dietary Exposure Assessment of Fipronil in Taiwan

The dietary exposure assessment of fipronil was achieved by simulating the dietary profiles of 200,000 individuals in Taiwan. The age-stratified (age 0–100) and sex-stratified demographic data were obtained from the Department of Household Registration, Ministry of Interior (data retrieved from April 2023). The body weight’s age- and sex-stratified data (mean and standard deviation) was acquired from the Nutrition and Health Survey in Taiwan (NAHSIT, 2013–2016). The simulated number of 200,000 was set to meet the minimal sample size of 10 for each subgroup (per sex per age). The food intakes of 11 food categories (Table 1) were organized from the National Food Consumption Database [31], a four-level food categorization system that provides food consumption estimates for the general population or consumer-only.

Truncated normal distribution was used to randomly assign the body weight and food intakes *(Food intake_i_)* for the simulated male (*n* = 98,788) and female (*n* = 101,212) individuals. Fipronil residues in food *(Residue_i_)* were stochastically generated using the transformed posterior mean and standard deviation (Formulas (1) and (2)), following lognormal distribution.

(1) $$\mathrm{Meanlog}=\log(\frac{mean^{2}}{\sqrt{SD^{2}+mean^{2}}})$$

(2) $$\mathrm{SDlog}=\sqrt{\log\left(1+\frac{SD^{2}}{mean^{2}}\right)}$$

where Meanlog and SDlog are the mean and standard deviation of the distribution on the log scale. Finally, the aggregate exposure of fipronil was calculated using the following Equation (3):

(3) $$\mathrm{Aggregate}\;\mathrm{fipronil}\;\mathrm{exposure}\;\left(\frac{\mathrm{mg}}{\mathrm{kg}}/\mathrm{day}\right)=\sum_{i=a}^{k}\frac{food\;intake_{i,j}\left(\frac{g}{day}\right)\times Residue_{i,\;j}\left(ppm\right)}{bodyweight_{j}\;\left(kg\right)\times 1000}$$

where *a*-*k* denotes the 11 food categories, *j* = 1,…, The lower bound (LB, general population) and upper bound (UB, consumer only) estimates were reported accordingly.

### 2.2. Revisit the Health Reference Dose of Fipronil Using Bayesian Benchmark Dose Modeling

The probabilistic reference dose (pRfD) of fipronil was derived using a web-based system, Bayesian BenchMark Dose modeling (BBMD, Available online: https://benchmarkdose.com/ accessed on 4 July 2023) [28]. The chronic animal data of fipronil were retrieved from the toxicological evaluation report for pesticide residues in food in 2021 [32]. Convulsions in male SD rats were deemed as the most sensitive endpoint and subject to probabilistic dose-response assessment (dose: 0, 0.019, 0.059, 1.27, and 12.68 mg/kg/day; incidence/total animals: 0/50, 0/50, 3/50, 1/50, and 5/50). The default models for dichotomous data (Logistic, Loglogistic, Probit, Logprobit, Quantal linear, Multistage (2nd order), Weibull, and Dichotomous Hill) and non-informative prior were used to fit the dose-response data, using Markov chain Monte Carlo (MCMC) simulation. Three Markov chains were calculated with 50,000 iterations per chain. The model distributions were estimated using the last 75,000 iterations (25,000 iterations per chain). The random seed was set at 76,316. From the fitted dose-response curves, BMD distributions were acquired using 10% extra risk as the benchmark response (BMR). The model average from 8 dichotomous models using the posterior model weights was used to establish the BMD distribution.

Next, BMD in the rat (BMD_rat_) were converted to BMD in human (BMD_h_), using the following parameters: human body weight, 70 kg; allometric scaling exponent (mean), 0.7; allometric scaling exponent (standard deviation), 0.0243. We applied probabilistic distribution to describe the uncertainty in inter-species (geometric mean (GM) = 1; geometric standard deviation (GSD) = 1.95) and intra-species (GM = 0.746; GSD = 1.5935) extrapolation. The estimated human dose, where 50% of the population has effects greater than or equal to 10% extra risk (HD^0.5^), the estimated human dose at which 1% population has effects greater than or equal to 10% extra risk ($HD_{M=0.1}^{I=0.01})$, and probabilistic reference dose (pRfD, the 5th percentile $HD_{M}^{I}$) were reported accordingly.

### 2.3. Risk Characterization of Fipronil Exposure in Taiwan

The fipronil-induced health risk in Taiwan was characterized using a probabilistic hazard quotient (HQ) approach. Briefly, the HQ or probabilistic HQ (pHQ) was calculated by dividing the aggregate fipronil exposure by the ADI or the probabilistic reference dose (pRfD, the 5th percentile of $HD_{M=0.1}^{I=0.05}$):

(4) $$\left(p\right)\mathrm{HQ}=\frac{Aggregate\;fipronil\;exposure\;\left(\frac{\frac{mg}{kg}}{day}\right)}{ADI\;or\;pRfD\;\left(\frac{\frac{mg}{kg}}{day}\right)}$$

### 2.4. Statistical Analysis and Data Visualization

The MCMC runs and the simulation for the Taiwanese population were performed in R software (version 4.0.4, R Development Core Team, Vienna, Austria), using packages “rstan (version 2.21.2)” and “truncnorm (version 1.0-9)”. The bar graphs, line graphs, and scatter plots were created using GraphPad Prism 9 (version 9.5.0).

## 3. Results

### 3.1. Fipronil Residues in Food

**Figure 1:**
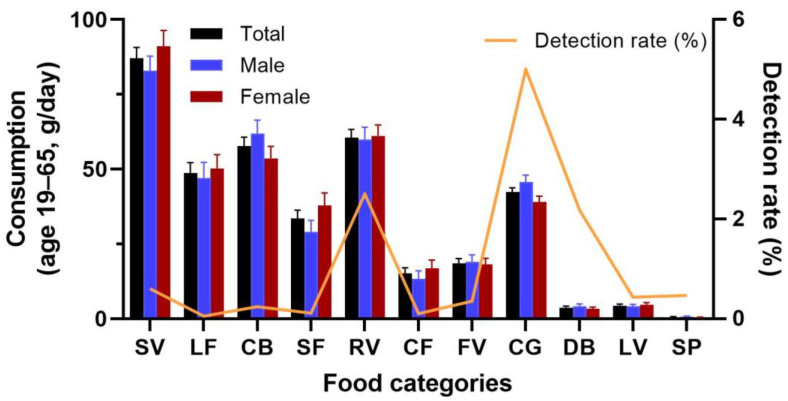
Comparative analysis of food intake and detection rate for each food category.

Among the investigated food categories, the detection rate of fipronil was 0.05% for LF, 0.1% for CF, 0.11% for SF, 0.24% for CB, 0.35% for FV, 0.43% for LV, 0.47% for SP, 0.6% for SV, 2.18% for DB, 2.5% for RV, and 5% for CG (chicken egg, CG, Table 1). The detection limit was 0.002 ppm. The highest concentration was found in mustard green (1.69 ppm). The root and stem vegetables and chicken eggs are likely the major contributors to total fipronil exposure, considering both the intake and detection rates of fipronil in food (Figure 1).

### 3.2. Reconstruction of Fipronil Residues Using MCMC Simulation

**Figure 2:**
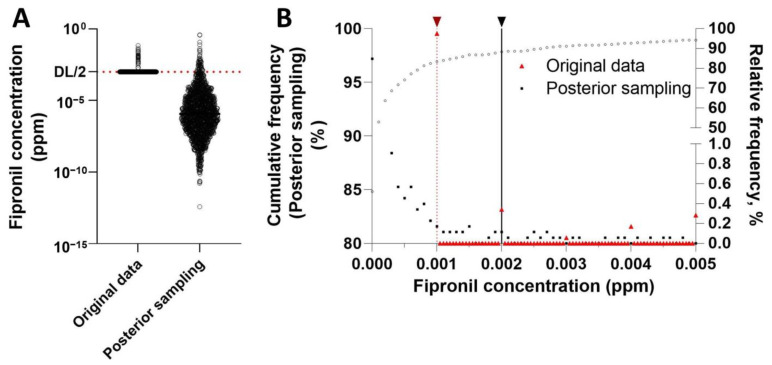
Reconstruction of fipronil residues using MCMC simulation: an example of root and stem vegetables (RV). (**A**) A scatter plot illustrates the difference between the original data and the data sampled from the posterior distribution of fipronil residues in root and stem vegetables (*n* =1760). In the original dataset, samples with non-detect values were shown as half of the detection limit (DL/2, *n* =1716). (**B**) Comparative analysis of the original data (red triangle) and the data sampled from the posterior distribution (black square), using a cumulative frequency plot (open circle, left y axis) and a relative frequency plot (right y axis). The black solid line denotes the detection limit (0.002 ppm) and the red dash line indicates the half of the detection limit (0.001 ppm).

Using MCMC simulation, we reconstructed the lognormal distribution of fipronil residues in food. Figure 2 illustrates the reconstruction of fipronil residue distribution in RV. The fipronil concentrations of the samples below the detection limit (*n =* 1716) were replaced by the random values generated from the posterior distribution (Figure 2A). This approach kept the same proportion of detectable samples in the original dataset (2.5%) yet projected fipronil concentrations in non-detects, following the lognormal distribution (Figure 2B).

### 3.3. Probabilistic Exposure Assessment of Fipronil in Taiwan

**Figure 3:**
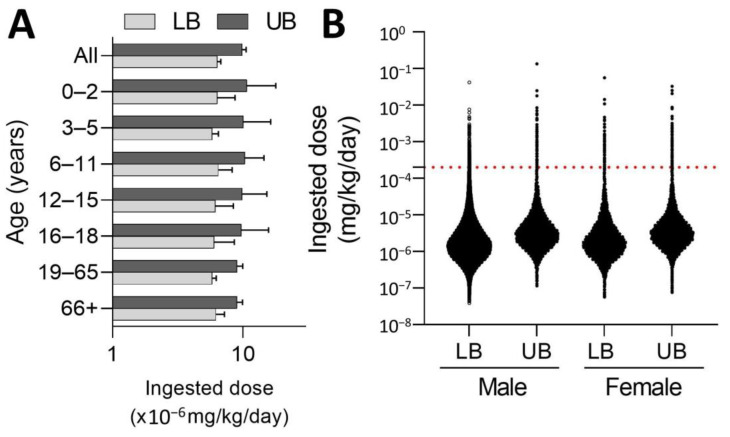
**Probabilistic exposure assessment of fipronil in Taiwan.** The lower bound (LB) and upper bound (UB) exposure estimates are summarized by (**A**) age (all ages, toddlers (0–2 years old), preschoolers (3–5 years old), children (6–11 years old), pre-teenagers (12–15 years old), teenagers (16–18 years old), adults (19–65 years old), and elderly (≧66 years old)) or (**B**) sex. The red dash line indicates the acceptable daily intake (ADI) of fipronil (0.0002 mg/kg/day) proposed by the Joint FAO/WHO Meeting on Pesticide Residues (JMPR).

The estimated exposure of fipronil to the general public ranges from 6.38 × 10^−6^ ± 0.00017 mg/kg/day (LB) to 9.83 × 10^−6^ ± 0.00034 mg/kg/day (UB), where toddlers (age 0–2 years old) and preschoolers (age 3–5 years old) have slightly higher fipronil exposures than other age groups (Figure 3A). There is no significant difference in fipronil exposures between males (LB: 6.2 × 10^−6^ mg/kg/day; UB: 10.5 × 10^−6^ mg/kg/day) and females (LB: 6.5 × 10^−6^ mg/kg/day; UB: 9.2 × 10^−6^ mg/kg/day, Figure 3B), where less than 0.35% of the males (0.23–0.34%) or females (0.22–0.28%) reported a fipronil exposure greater than the deterministic acceptable daily intake of fipronil (0.0002 mg/kg/day).

### 3.4. Probabilistic Dose-Response Assessment of Fipronil

**Figure 4:**
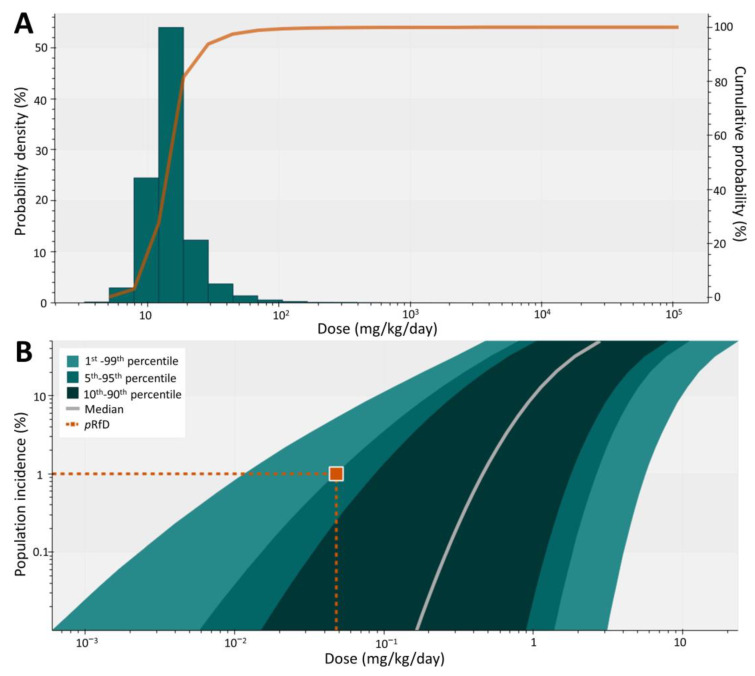
**Probabilistic dose-response assessment of fipronil.** (**A**) The probability density and cumulative probability distribution of BMDL_10_ and (**B**) the probabilistic $HD_{M}^{I}$ plot for fipronil-induced convulsion in male SD rats. The probabilistic reference dose (pRfD) is defined as the 5th percentile $HD_{M=0.1}^{I=0.01}$, representing the estimated human dose, where the population has 1% incidence of the target magnitude of effect (BMR = 10%).

The BMDL_10_ values were sampled from the posterior distributions (*n =* 8) with their corresponding model weights for Logistic (20.5%), LogLogistic (8.9%), Probit (20.7%), LogProbit (7.5%), Quantal linear (20.1%), Multistage (8.7%), Weibull (9.5%), and Dichotomous Hill (4.1%) (Figure 4A). The BMDL_10_, the estimated human dose, where 50% of the population has effects greater than or equal to 10% extra risk (HD^0.5^), and the dose at which 1% population has effects greater than or equal to 10% extra risk ($HD_{M=0.1}^{I=0.01})$ were calculated using Bayesian benchmark dose analysis (Table 2). Our risk assessment goal was to protect 99% of the population with effects less than 10% extra risk; therefore, the pRfD was set at 0.048 mg/kg/day (Figure 4B).

**Probabilistic risk characterization of fipronil in Taiwan**

**Figure 5:**
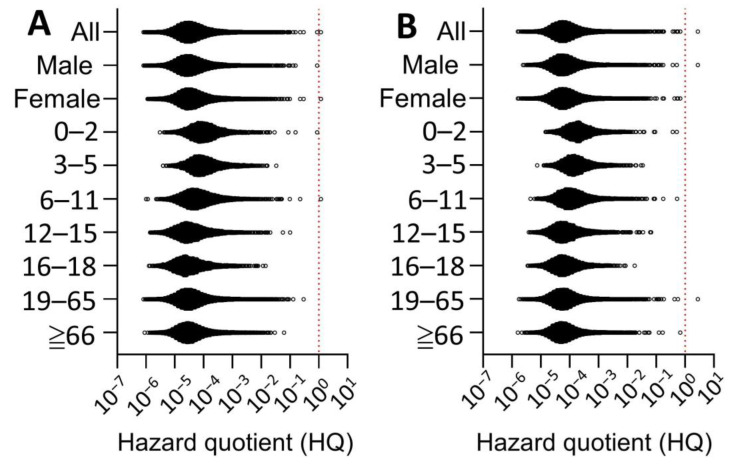
Probabilistic risk characterization of fipronil using lowerbound (**A**) and upperbound (**B**) exposure estimates in Taiwan. The dash line indicates the margin of safety (HQ = 1).

Next, the pRfD was used to characterize the chronic health risk caused by fipronil, adopting a hazard quotient approach (Figure 5). Regardless of LB or UB exposure scenarios, only one out of the 200,000 simulated individuals reported fipronil exposure exceeding the pRfD value (i.e., HQ > 1). Overall, younger individuals receive greater risks than the elderly.

## 4. Discussion

The importance of addressing uncertainty and variability in exposure and dose-response assessments has been highlighted by the World Health Organization International Programme on Chemical Safety (WHO/IPCS) since 2008 [33,34,35]. Current practices in fipronil risk assessment have often considered the uncertainty and variability in exposure assessments [20,36,37], yet there is no study performing probabilistic dose-response analysis for the risk characterization. To the best of our knowledge, this study is the first to characterize the fipronil-induced health risk by adopting both probabilistic exposure assessment and probabilistic dose-response evaluation. This study brings several refinements and essential insights into the dietary exposure assessment, probabilistic dose-response analysis, and risk characterization of fipronil in Taiwan.

We significantly reduced the uncertainty in the dietary exposure assessment of fipronil. The uncertainty in exposure assessment usually results from an insufficient understanding of relevant exposure scenarios, exposure models, and model inputs [33]. Regarding dietary exposure assessment, uncertainties can arise from food consumption estimates or chemical residues in food. The uncertainty associated with food consumption was minimized via conducting lower bound (i.e., the general public) or upper bound (i.e., consumer only) exposure estimations. However, the actual concentrations of non-detects can range from zero to the analytical detection limit (DL), with which the uncertainty is difficult to address. Investigators often tackle the non-detects using a conservative approach: substituting the non-detects with $\frac{DL}{2}$, $\frac{DL}{\sqrt{2}}$, or DL, yet simply substituting values for non-detects has been shown to cause biased results [38]. Alternative approaches include robust regression on order statistics (i.e., robust ROS), maximum likelihood estimation (MLE), and Kaplan—Meier (KM) method [39]. Most methods can provide a reliable estimation when the censoring rate is less than 50% but are inapplicable when censoring rates are greater than 80%. Bayesian inference has been increasingly used to reconstruct the sample distribution for datasets with high censoring rates (>80%) in the context of risk assessment [30,40,41]. In this study, the censoring rates of fipronil concentrations (i.e., below DL) in food are generally greater than 95%. Therefore, we adopted the Bayesian MCMC approach to reduce the uncertainty deriving from the non-detects.

The selection of prior distribution in MCMC modeling could significantly influence the results of the posterior distribution. Empirically, the chemical residues in food are often assumed to follow a normal or lognormal distribution. In this study, we conducted normality tests (i.e., Kolmogorov—Smirnov and Shapiro—Wilk test) for the fipronil concentrations in fruits and vegetables (*n =* 118), confirming that the data follow lognormal distribution rather than the normal distribution. Our modeled data fit very well with the original data, as determined by the cumulative and relative frequency plots (Figure 2B). Compared with the simple substitution method, the sample distribution reconstructed using MCMC does largely decrease the uncertainty associated with non-detects.

Herein, we refined the dose-response analysis to derive the exposure limit of fipronil. Probabilistic dose-response assessment is a relatively new concept incorporated only in a few studies [25,29,42,43]. The unified framework for probabilistic dose-response assessment was developed in 2015, where the lower bound fifth percentile estimate of “target human dose” ($HD_{M}^{I})$, instead of the traditional RfD or ADI, was proposed to serve as the exposure limit for risk characterization [23]. Target human dose requires the declaration of the magnitude of critical effect M and target population incidence I. Instead of applying deterministic uncertainty factors in the conventional approach, $HD_{M}^{I}$ incorporates probabilistic factors for interspecies body weight scaling, interspecies toxicokinetic and toxicodynamic differences, and human variability for population incidence I. The lower bound $HD_{M}^{I}$ estimate, or pRfD, is scientifically rigorous and transparent, informing risk management for different decision contexts [25]. In this study, $HD_{M=0.1}^{I=0.01}$ was calculated to represent the dose at which 1% population has effects greater than or equal to 10% extra risk. In other words, we aimed to protect 99% of the people from the fipronil-induced neurotoxicity of 10% extra risk. Likewise, we can easily tailor the pRfD with flexible M and I for different decision-making contexts, such as benefit-cost analysis, life-cycle impact analysis, and emergency responses to environmental incidents.

The ADI set by JMPR (0.0002 mg/kg/day) for fipronil is more conservative than the pRfD derived in this study, which is concordant with the results from the previous analysis on 1522 chemicals and endpoints [25]. From the BBMD analysis, approximately 12.6% of the uncertainty is associated with the POD, 57.9% for human variability, 28.4% for interspecies difference, and 1.1% for allometric scaling. The moderate uncertainty associated with POD indicates that the BMDL estimations from the eight models are similar, reporting BMDL estimates from one to 100 mg/kg/day. Overall, the BMDL values determined using BBMD are about 100-fold greater than the NOAEL of fipronil-induced neurotoxicity in male rats, resulting in a larger pRfD than the ADI.

In this study, we conclude that the health risk caused by fipronil exposure in Taiwan is minimal. Less than 0.4% of the population had LB or UB exposure estimates exceeding the traditional ADI. Additionally, only one out of the 200,000 simulated individuals reported a pHQ value greater than one, regardless of the LB or UB exposure scenario. In a closer look, this simulated individual is a 38 years-old male with a body weight of 74.1 kg. He was subject to a highly unusual exposure scenario, having a relatively high RV consumption (258 g/day) and an extremely high concentration of fipronil residue in RV. Nevertheless, the relatively high detection rates of fipronil in CG and RV necessitate continuous post-market investigation in the future.

This study is not without limitations. Firstly, the food consumption data were based on a 24-hour dietary recall questionnaire. Recall bias and misrepresentation of portion sizes may occur while collecting food consumption data. However, we perform the best-case (LB) and the worst-case (UB) exposure assessments to better describe the uncertainty in food consumption data. Secondly, the sample size of CG in fipronil analysis is relatively small (*n =* 40) compared with other fruits and vegetables. Continuous sampling and determination of fipronil concentrations in CG items are warranted. Lastly, we applied the default probabilistic factors to describe human variability in toxicokinetics and toxicodynamics (GM = 0.746, GSD = 1.59), which may underestimate or overestimate the “true” inter-individual variability in fipronil’s toxicokinetics and toxicodynamics. If accessible, CASF is highly recommended to replace the default probabilistic factor accounting for human variability. Notwithstanding these limitations, this case study demonstrated the utility of Bayesian inference in reducing the uncertainty in exposure assessment and dose-response analyses. We strongly advocate for adopting a probabilistic framework in the dietary risk assessments of foodborne chemicals.

## Figures and Tables

**Table 1:** Summary of fipronil residual concentration in food.

Food Category	Abbreviation	Food Items	Total	Detects	Conc.	Std.
Large berry fruit	LF	dragon fruit, pineapple, banana, sugar apple, papaya, passion fruit, avocado, caimito, aiyu, rambutan, kiwi fruit, star apple, jack fruit, and canistel fruit	2042	1	0.003	-
Small berry fruit	SF	guava, grape, wax apple, strawberry, fig, carambola, jabuticaba, and mulberry	923	1	0.002	-
Citrus fruit	CF	pomelo, orange, madarin, tangerine, lemon, lime, grapefruit, and kumquat	1046	1	0.014	-
Cabbage	CB	broccoli, cabbage, garden lettuce, and kohlrabi	1265	3	0.229	0.138
Small leafy vegetable	SV	bok choy, water spinach, rape, pak choy, lettuce, basil, spinach, celery, leaf of sweat potato, chives, kale, mustard, chayote vine, bird-nest fern, scallion, Ganges amaranth, Japanese mustard spinach, garlic, Okinawa spinach, chrysanthemum greens, Malabar spinach, garland chrysanthemum, white water, vegetable fern, white mugwort, Rangoon ceeper, shallot, purslane, jute, beefsteak plant, aloe, and Chinese violet	6974	42	0.115	0.328
Fruit vegetables	FV	tomato, eggplant, bell pepper, baby corn, chili, okra, and roselle	2556	9	0.018	0.025
Legume vegetables	LV	snap bean, kidney bean, french bean, navy bean, string bean, garden pea, sugar pea, snow pea, and lima bean	1867	8	0.010	0.005
Root and stem vegetables	RV	carrot, bamboo, potato, sweet potato, onion, taro, turnip, water bamboo, common yam, ginger, asparagus, Arctium, garlic, lotus root, water chestnut, beetroot, and cassava	1760	44	0.013	0.013
Dry beans	DB	soybean, peanut, mung bean, adzuki bean, jackfruit seed, yardlong bean, sunflower seed, rapeseed, sunflower seed, cottonseed, hyacinth bean, lotus seed, sesame seed	229	5	0.006	0.004
Chicken egg	CG	chicken egg	40	2	0.014	0.011
Spice	SP	butterfly pea flower, goji berry root, Chinese celery, mesona, star anise, chamomile, rose, mugwort, chia seeds, Sichuan peppercorn, and mint	858	4	0.051	0.027

**Table 2:** Summary statistics of BMDL_10_, HD^0.5^, and $HD_{M=0.1}^{I=0.01}$.

Unit: mg/kg/day	BMDL_10_	HD^0.5^	$HD_{M=0.1}^{I=0.01}$	ADI
Median	13.48	2.80	0.46	0.0002
90th percentile	9.96	1.06	0.088	
95th percentile	8.67	0.80	0.048 ^†^	
99th percentile	6.49	0.48	0.012	
Mean	22.21	4.96	1.03	
Standard deviation	107.37	107.37	39.58	

^†^ The pRfD is defined as the 95th percentile $HD_{M=0.1}^{I=0.01}.$

## Data Availability

Publicly available datasets were analyzed in this study. This data can be found here: https://data.gov.tw/dataset/8935, accessed on 4 July 2023. The exposure assessment data are available in Supplementary Materials. The dose-response modeling results are available at https://benchmarkdose.com/run/2603a51c-be51-46a6-bc6c-cf3e5a1d2d99/, accessed on 4 July 2023.

## Supplementary Materials

The following supporting information can be downloaded at: https://www.mdpi.com/article/10.3390/toxics11080677/s1, File S1: The R code for reconstructing the sample distribution; File S2: The exposure assessment of fipronil in Taiwan (Lower bound); File S3: The exposure assessment of fipronil in Taiwan (Upper bound).

## Abbreviations

Acceptable daily intake	ADI
Bayesian Benchmark dose	BBMD
Benchmark dose lower confidence limit	BMDL
Chemical-specific adjustment factor	CASF
Cabbage	CB
Chicken egg	CG
Citrus fruit	CF
Dry bean	DB
Fruit vegetable	FV
Hazard quotient	HQ
Large berry fruit	LF
Legume vegetable	LV
Lower-bound exposure	LB
Markov Chain Monte Carlo simulation	MCMC
No-observed adverse effect level	NOAEL
Point of departure	POD
Probabilistic reference dose	pRfD
Root and stem vegetable	RV
Small berry fruit	SF
Small leafy vegetable	SV
Spice	S
Upper-bound exposure	UB
